# Probing long-lived radioactive isotopes on the double-logarithmic Segrè chart

**DOI:** 10.3389/fchem.2024.1057928

**Published:** 2024-02-12

**Authors:** Haitao Shang

**Affiliations:** Institute of Ecology and Evolution, University of Oregon, Eugene, OR, United States

**Keywords:** non-radioactive elements, radioactive elements, stable isotopes, long-lived isotopes, unknown isotopes, power laws, double-logarithmic scale, Segrè chart

## Abstract

Isotopes have been widely applied in a variety of scientific subjects; many aspects of isotopes, however, remain not well understood. In this study, I investigate the relation between the number of neutrons (*N*) and the number of protons (*Z*) in stable isotopes of non-radioactive elements and long-lived isotopes of radioactive elements at the double-linear scale (conventional Segrè chart) and the double-logarithmic scale. Statistical analyses show that *N* is a power-law function of *Z* for these isotopes: *N* = 0.73 × *Z* ^1.16^. This power-law relation provides better predictions for the numbers of neutrons in stable isotopes of non-radioactive elements and long-lived isotopes of radioactive elements than the linear relation on the conventional Segrè chart. The power-law pattern reveled here offers empirical guidance for probing long-lived isotopes of unknown radioactive elements.

## 1 Introduction

Isotopes are variants of elements that possess the same number of protons but differ in the number of neutrons (De Groot, 2004; Ellam, 2016). Several hundred isotopes have been detected in natural environments on Earth, while thousands of other isotopes are continuously created in various areas in the universe (White, 2014; McSween Jr and Huss, 2022). Since the first discovery by Frederick Soddy in 1913 (Soddy, 1913), isotopes have been widely applied in different subjects. For example, ecologists use isotopic signatures to study the exchanges of materials between life and environments (Michener and Lajtha, 2008), biologists investigate metabolic processes on the basis of intermolecular and intramolecular isotopic effects (Kohen and Limbach, 2005), and geochemists use the fingerprints that isotopes left in sedimentary records to reconstruct the evolutionary trajectories of life and environments on the ancient Earth (White, 2014).

Isotopes are classified into two categories according to their stability: stable and radioactive isotopes. Stable isotopes of one element do not transform into other elements under natural conditions, while radioactive isotopes decay to other elements after certain time periods with specific half-lives (De Groot, 2004; Ellam, 2016). Among the known 118 elements on the periodic table, 80 of them have one or more stable isotopes and are called as non-radioactive elements (De Groot, 2004; Ellam, 2016). This non-radioactive category includes the elements with atomic number (*Z*) less than 83 except for two elements that have no stable isotopes (i.e., technetium with *Z* = 43 and promethium with *Z* = 61) (De Groot, 2004; Ellam, 2016). In contrast, nature creates neutrons and protons in an asymmetric manner in the nuclei of elements with atomic number larger than 83; these elements are also referred to as radioactive elements (Blatt and Weisskopf, 1991; Thomson, 2013). As the imbalance between neutrons and protons of a nucleus grows, its stability decreases; in this case, decay offers an chance for the nucleus to re-establish a balance between its neutrons and protons (Blatt and Weisskopf, 1991; Thomson, 2013). While radioactive elements eventually decay into other elements, their long-lived isotopes may have rather extended lifespans (Blatt and Weisskopf, 1991; Thomson, 2013). For example, the half life of ^209^Bi, an isotope of the radioactive element bismuth, is 2.01 × 10^19^ years, which is 1.5 × 10^9^ times greater than the age of the universe (De Groot, 2004; Ellam, 2016).

Combining protons and neutrons in an arbitrary way does not necessarily generates a stable nucleus. A nucleus is unbounded when it is outside the valley of stability, which is defined by the neutron drip line and proton drip line (Hansen, 1993; Thoennessen, 2004). Substantial efforts have been dedicated to exploring the principles and mechanisms for the stability of isotopes. Since Myers, Światecki, Viola Jr., and Seaborg predicted that nuclei of superheavy elements occupy a region called as stability island on the Segrè chart (a plot arranging nuclides by proton number *Z* and neutron number *N* at the double-linear scale) (Myers and Swiatecki, 1966; Viola Jr and Seaborg, 1966), the concept of the “island of stability” has been a dominant paradigm in the study of superheavy nuclei. A variety of intriguing properties in the island of stability have been revealed. For example, it was discovered in the late 1940s that nuclei possessing a magic number (2, 8, 20, 28, 50, 82, or 126) of protons or neutrons exhibit much higher stability than other nuclei; this phenomenon then became the basis of the nuclear shell model (Wigner, 1937; Mayer, 1948; Haxel et al., 1949; Kanungo et al., 2002).

Here, I investigate the relation between *N* and *Z* in stable isotopes of non-radioactive elements and long-lived isotopes of radioactive elements at double-linear and double-logarithmic scales and show that *N* is a power-law function of *Z* in these isotopes. The power law refers to a functional relation between two variables in which one variable changes as a power of the other. On a double-logarithmic plot, the power law appears as a straight line, implying that the underlying regularity of this relation is independent of the specific scales one investigates (Clauset et al., 2009; Alstott et al., 2014). Statistical analyses in this work demonstrate that the power-law relation on the double-logarithmic Segrè chart provides more accurate predictions for *N* than the linear relation obtained on the conventional double-linear Segrè chart. These results offer new insights into the future searching for the long-lived isotopes of unknown radioactive elements.

## 2 Data and methods

The dataset on isotopes is from Nubase 2020 (Kondev et al., 2021). Except for technetium and promethium, each non-radioactive element has one or more stable isotopes; the numbers of neutrons in these isotopes usually are close to one another. For each non-radioactive element, I calculate the average number of neutrons in its stable isotopes and denote this mean value by $\bar{N}$ . On the other hand, the lifespans of a radioactive element’s isotopes often vary across a large range. For each radioactive element, I take the isotope with the longest lifespan as the representative long-lived isotope and denote the number of neutrons in this representative isotope by *N*_rad_. For simplicity, I henceforth use *N* to denote $\bar{N}$ or *N*_rad_.

**FIGURE 1:**
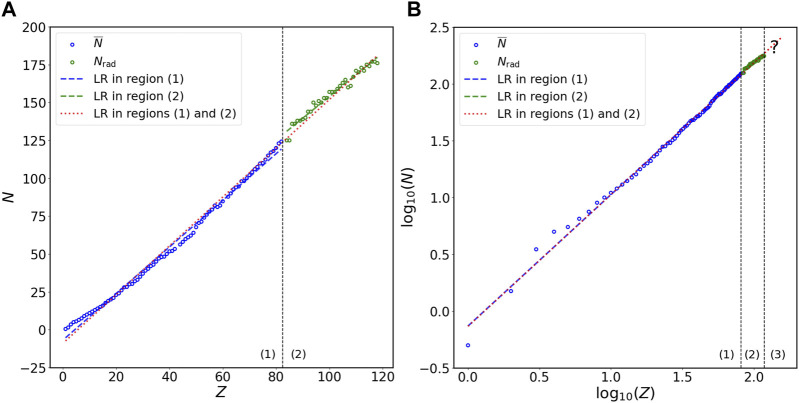
Relation of *N* versus *Z* in stable isotopes of non-radioactive elements and long-lived isotopes of radioactive elements at the **(A)** double-linear and **(B)** double-logarithmic scales. The *N* on the vertical axis represents either $\bar{N}$ (in region (1)) or *N*_rad_ (in regions (2) and (3)). Region (1) in panel **(A)** or **(B)** is for $\bar{N}$ versus *Z* in stable isotopes of non-radioactive elements (blue circles), region (2) in panel **(A)** or **(B)** is for *N*_rad_ versus *Z* in long-lived isotopes of radioactive elements (green circles), and region (3) in panel **(B)** is for long-lived isotopes of unknown radioactive elements. Blue line in region (1) of panel **(A)** or **(B)** is the linear regression (LR) for stable isotopes of non-radioactive elements (blue circles) at the linear or logarithmic scale, respectively. Green line in region (2) of **(A)** or **(B)** is the LR for long-lived isotopes of radioactive elements (green circles) at the linear or logarithmic scale, respectively. Red line in regions (1) and (2) of panel **(A)** or **(B)** is the LR for both stable isotopes of non-radioactive elements and long-lived isotopes of radioactive elements at the linear or logarithmic scale, respectively. Red line (with question mark) in region (3) of panel **(B)** represents the predictions for *N* values of long-lived isotopes of unknown radioactive elements. The results of statistical analyses are presented in Tables 1, 2. The magnified plots for panels **(A)** and **(B)** are presented in Figures 2, 3, respectively.

**FIGURE 2:**
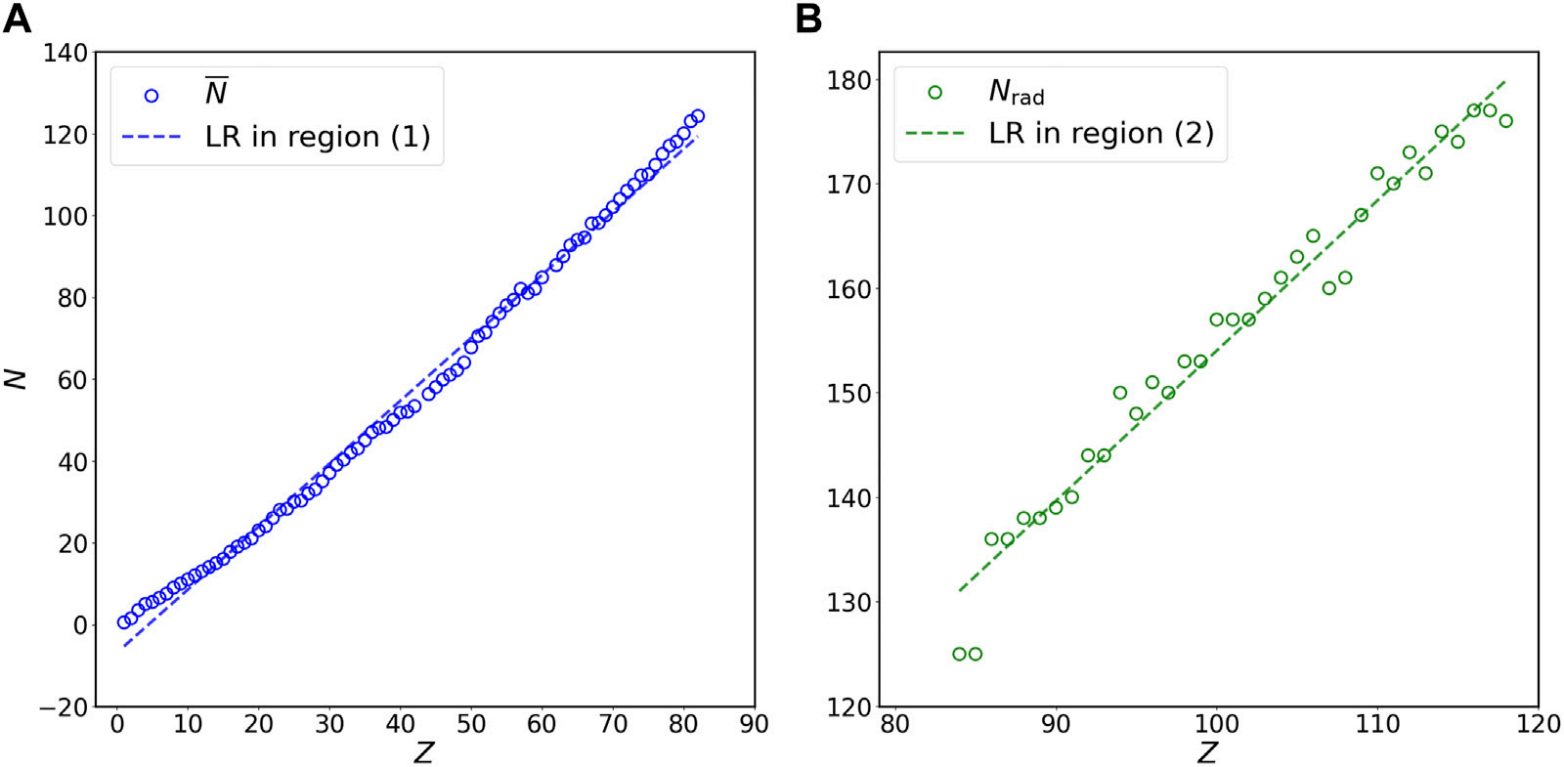
Relation of *N* versus *Z* for **(A)** stable isotopes of non-radioactive elements (blue circles) and **(B)** long-lived isotopes of radioactive elements (green circles) at the double-linear scale. Panels **(A)** and **(B)** in this figure are the magnification of region (1) and region (2) in Figure 1A, respectively. The *N* on the vertical axis represents $\bar{N}$ for panel **(A)** and *N*_rad_ for panel **(B)** in this figure. Blue and green lines are the linear regression (LR) for stable isotopes of non-radioactive elements (blue circles) and long-lived isotopes of radioactive elements (green circles), respectively, at the double-linear scale. The results of statistical analyses are presented in Tables 1, 2.

**FIGURE 3:**
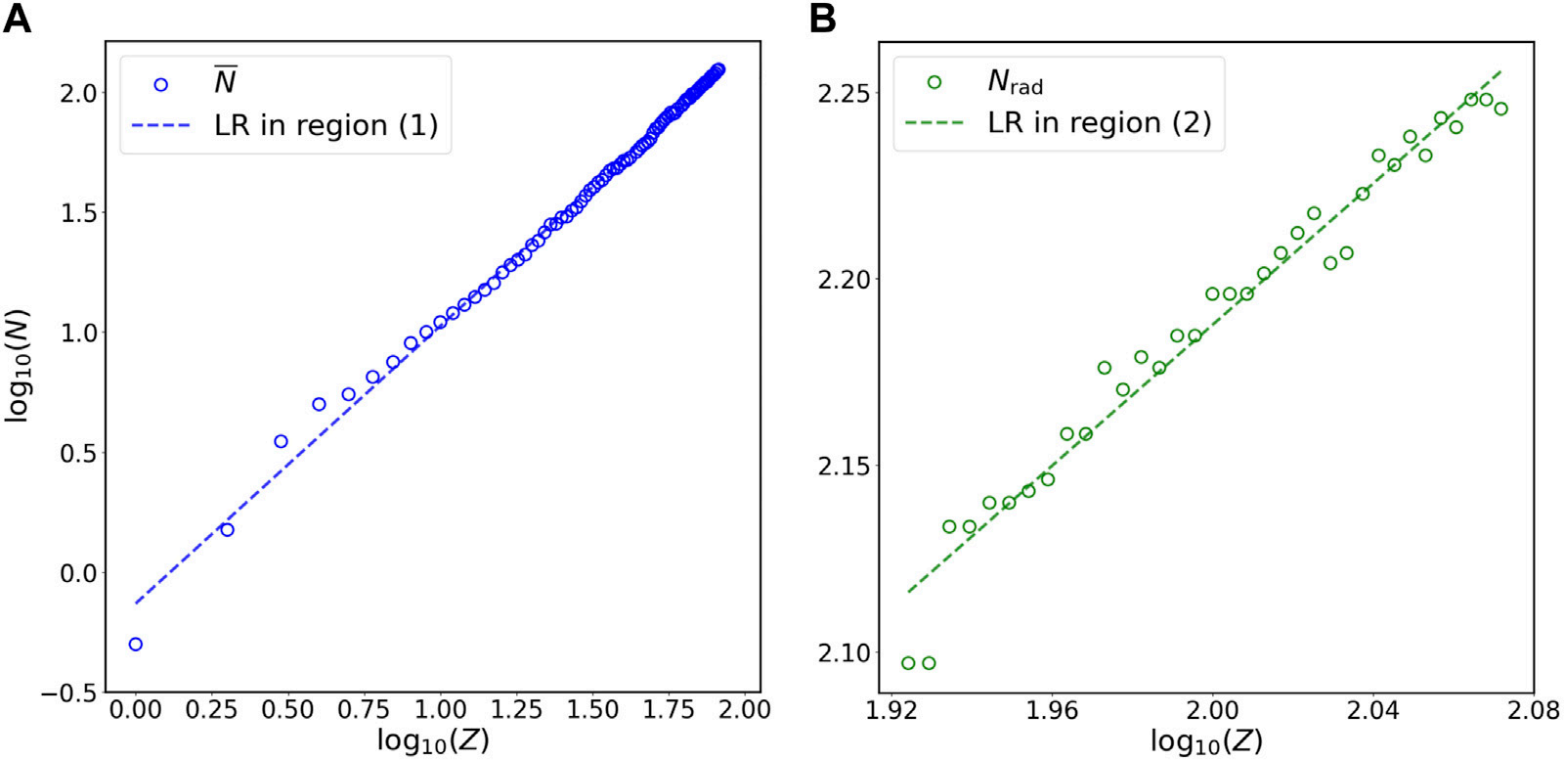
Relation of *N* versus *Z* for **(A)** stable isotopes of non-radioactive elements (blue circles) and **(B)** long-lived isotopes of radioactive elements (green circles) at the double-logarithmic scale. Panels **(A)** and **(B)** in this figure are the magnification of region (1) and region (2) in Figure 1B, respectively. The *N* on the vertical axis represents $\bar{N}$ for panel **(A)** and *N*_rad_ for panel **(B)** in this figure. Blue and green lines are the linear regression (LR) for stable isotopes of non-radioactive elements (blue circles) and long-lived isotopes of radioactive elements (green circles), respectively, at the double-logarithmic scale. The results of statistical analyses are presented in Tables 1, 2.

To investigate the relation between *N* and *Z*, I first divide the plot of *N* versus *Z* into two regions (Figure 1): (1) non-radioactive elements ( $\bar{N}$ versus *Z*) and (2) radioactive elements (*N*_rad_ versus *Z*). I then apply linear regression to fit the data in region (1) and region (2) at the double-linear (Figure 1A) and double-logarithmic (Figure 1B) scales; blue and green lines in Figure 1 show the best-fitting lines in region (1) and region (2), respectively. The lines obtained at the double-linear scale (Figure 1A) are conventional linear regression lines, while the lines obtained at the double-logarithmic scale (Figure 1B) are referred to as power laws. The details of power-law analyses are described in Alstott et al. (2014) and Clauset et al. (2009). Moreover, to compare the effectiveness of predictions by the fitting formulas obtained using data in both regions (1) and (2) to that obtained only based on data in an individual region (1) or (2), I fit the data in both regions at the double-linear (Figure 1A) and double-logarithmic (Figure 1B) scales; red lines in Figure 1 show the best-fitting lines for all data in both regions (1) and (2). The magnified plots for Figures 1A, B are presented in Figure 2 and Figure 3, respectively.

To evaluate the goodness of fit of the fitting formulas, I calculate the coefficients of determination (*R* ^2^’s) and perform the Kolmogorov-Smirnov (KS) test (Massey Jr, 1951) and Cramér-von Mises (CM) two-sample test (Anderson, 1962). The metric *R* ^2^ measures the fraction of variations in a dependent variable that can be explained by a fitting function; a larger value of *R* ^2^ indicates a better fitting and therefore a more reliable model (Freund and Wilson, 2003). The KS statistic is defined as $\sup\left|M_{\text{m}}-M_{\text{d}}\right|$ , which measures the largest distance between the cumulative distribution functions (CDFs) of a distribution that best fits the data (*M*_m_) and the data themselves (*M*_d_) (Massey Jr, 1951). The CM test is defined as $\frac{A\times B}{{\left(A+B\right)}^{2}}\left[\sum_{a=0}^{A}{\left(M_{\text{m}}\left(x_{a}\right)-M_{\text{d}}\left(x_{a}\right)\right)}^{2}+\sum_{b=0}^{B}{\left(M_{\text{m}}\left(x_{b}^{\prime }\right)-M_{\text{d}}\left(x_{b}^{\prime }\right)\right)}^{2}\right]$ , where ${\left\{x_{a}\right\}}_{a=0}^{A}$ and ${\left\{x_{b}^{\prime }\right\}}_{b=0}^{B}$ are samples independently drawn from two distributions with CDFs *M*_m_ and *M*_d_, respectively (Anderson, 1962). To perform the KS and CM tests, I set the null hypothesis and two-sided alternative as *H*_0_: *M*_m_ = *M*_d_ and *H*_1_: *M*_m_ ≠ *M*_d_, respectively. The critical *p*-value for these tests is set to 0.05; *p* > 0.05 suggests that a function fits the data well while *p* ≤ 0.05 suggests that a function does not adequately describes the data. To compare the effectiveness of predictions by the fitting formulas obtained at the double-linear and double-logarithmic scales, I calculate the average relative errors (AREs) and root mean square errors (RMSEs) for the predicted values of these fitting formulas; a smaller value of ARE or RMSE indicates a better model.

## 3 Results

Figures 1A, B show the linear regression for $\bar{N}$ or *N*_rad_ versus *Z* at the double-linear and double-logarithmic scales, respectively. Region (1) in these two panels presents the dataset on stable isotopes of non-radioactive elements (blue circles) and the linear regression line (blue line) for these data, while region (2) illustrates the dataset on long-lived isotopes of radioactive elements (green circles) and the linear regression line (green line) for these data. The magnified plots for Figures 1A, B are illustrated in Figure 2 and Figure 3, respectively. The mathematical expressions for linear regression of the dataset on stable isotopes of non-radioactive elements (i.e., $\bar{N}$ versus *Z*) at the double-linear (Figure 2A) and double-logarithmic (Figure 3A) scales are *N* = 1.54 × *Z* − 6.86 and *N* = 0.74 × *Z* ^1.15^, respectively. For the dataset on long-lived isotopes of radioactive elements (i.e., *N*_rad_ versus *Z*), the mathematical expressions for linear regression at the double-linear (Figure 2B) and double-logarithmic (Figure 3B) scales are *N* = 1.44 × *Z* + 10.29 and *N* = 1.97 × *Z* ^0.95^, respectively. To evaluate how well these mathematical formulas fit the datasets, I calculate the coefficient of determination (*R* ^2^) and perform the KS and CM tests (Section 2); the statistical results are summarized in Table 1. All *R* ^2^ values are close to 1 and all *p*-values for the KS and CM tests are much greater than the critical value 0.05, indicating that all formulas presented in Table 1 fit the data in specific regions extraordinarily well.

**TABLE 1:** Coefficients of determination (*R* ^2^’s), *p*-values of the Kolmogorov-Smirnov test (*p*_KS_’s), and *p*-values of the Cramér-von Mises two-sample test (*p*_CM_’s) of the best-fitting formulas obtained in region (1), region (2), and both regions (1) and (2) in Figure 1 at the double-linear (Figure 1A) and double-logarithmic (Figure 1B) scales. Region (1) and region (2) in Figure 1A correspond to panel (A) and panel (B) in Figure 2, respectively; region (1) and region (2) in Figure 1B correspond to panel (A) and panel (B) in Figure 3, respectively.

Region in Figure 1	Panel in Figure 2 or Figure 3	Scale	Best-fitting line	Best-fitting formula	*R* ^2^	*p*_KS_	*p*_CM_
Region (1) in Figure 1A	Figure 2A	Linear	Blue	*N* = 1.54 × *Z* − 6.86	0.94	0.99	0.98
Region (1) in Figure 1B	Figure 3A	Logarithmic	Blue	*N* = 0.74 × *Z* ^1.15^	0.99	1.00	1.00
Region (2) in Figure 1A	Figure 2B	Linear	Green	*N* = 1.44 × *Z* + 10.29	0.96	0.98	0.99
Region (2) in Figure 1B	Figure 3B	Logarithmic	Green	*N* = 1.97 × *Z* ^0.95^	0.98	0.99	0.99
Regions (1) & (2) in Figure 1A	—	Linear	Red	*N* = 1.61 × *Z* − 9.01	0.98	0.99	0.99
Regions (1) & (2) in Figure 1B	—	Logarithmic	Red	*N* = 0.73 × *Z* ^1.16^	0.99	0.99	1.00

To test the effectiveness of predictions by the mathematical expressions in Table 1, I first use the fitting formulas for the dataset on stable isotopes of non-radioactive elements (i.e., $\bar{N}$ versus *Z*) at the double-linear (Figure 2A) and double-logarithmic (Figure 3A) scales to predict the values of $\bar{N}$ and *N*_rad_. The AREs and RMSEs of these predictions are presented in Table 2, which shows that switching from the fitting formula obtained at the double-linear scale (*N* = 1.54 × *Z* − 6.86) to the fitting formula obtained at the double-logarithmic scale (*N* = 0.74 × *Z* ^1.15^) reduces the AREs of the predictions for $\bar{N}$ by 86%, *N*_rad_ by 39%, and both $\bar{N}$ and *N*_rad_ by 84%, and reduces the RMSEs of the predictions for $\bar{N}$ by 56%, *N*_rad_ by 41%, and both $\bar{N}$ and *N*_rad_ by 42%. These results suggest that the power law offers more accurate predictions for *N* than the linear relation obtained at the double-linear scale. To further justify this implication, I use the linear regression formulas for the dataset on long-lived isotopes of radioactive elements (i.e., *N*_rad_ versus *Z*) at the double-linear scale (Figure 2B) and the double-logarithmic scale (Figure 3B) to predict the values of $\bar{N}$ and *N*_rad_. When using the power law (*N* = 1.97 × *Z* ^0.95^) instead of the linear relation (*N* = 1.44 × *Z* + 10.29), the AREs of the predictions for $\bar{N}$ , *N*_rad_, and both $\bar{N}$ and *N*_rad_ decrease by 62%, 13%, and 62%, respectively; the RMSEs of the predictions for $\bar{N}$ , *N*_rad_, and both $\bar{N}$ and *N*_rad_ decrease by 26%, 5%, and 25%, respectively (Table 2). These results also imply that the power law provides better predictions for *N* than the linear relation obtained at the double-linear scale.

**TABLE 2:** Average relative errors (AREs) and root mean square errors (RMSEs) of the predictions for *N* by the best-fitting formulas obtained in region (1), region (2), and both regions (1) and (2) in Figure 1 at the double-linear (Figure 1A) and double-logarithmic (Figure 1B) scales. The statistical results in the specific regions in Figure 1 from which the best-fitting formulas in this table are obtained is presented in Table 1. Region (1) and region (2) in Figure 1A correspond to panel (A) and panel (B) in Figure 2, respectively; region (1) and region (2) in Figure 1B correspond to panel (A) and panel (B) in Figure 3, respectively. The percentages in parentheses show how much the AREs or RMSEs of predictions for *N* in specific regions change when switching from linear relations to power laws; downward arrows indicate decreases.

Best-fitting formula	ARE of predictions in region (1)	ARE of predictions in region (2)	ARE of predictions in regions (1) & (2)	RMSE of predictions in region (1)	RMSE of predictions in region (2)	RMSE of predictions in regions (1) & (2)
*N* = 1.54 × *Z* − 6.86	29.61% (*↓* 86%)	4.54% (*↓* 39%)	21.98% (*↓* 84%)	2.82 (*↓* 56%)	7.47 (*↓* 41%)	4.75 (*↓* 42%)
*N* = 0.74 × *Z* ^1.15^	4.01%	2.52%	3.56%	1.25	4.43	2.74
*N* = 1.44 × *Z* + 10.29	87.02% (*↓* 62%)	1.43% (*↓* 13%)	60.91% (*↓* 62%)	13.51 (*↓* 26%)	2.55 (*↓* 5%)	11.36 (*↓* 25%)
*N* = 1.97 × *Z* ^0.95^	33.01%	1.24%	23.34%	9.98	2.43	8.46
*N* = 1.61 × *Z* − 9.01	39.35% (*↓* 90%)	2.37% (*↓* 11%)	28.43% (*↓* 88%)	3.39 (*↓* 59%)	4.34 (*↓* 17%)	3.46 (*↓* 23%)
*N* = 0.73 × *Z* ^1.16^	3.97%	2.11%	3.52%	1.39	3.61	2.65

To predict the *N* values of long-lived isotopes of unknown radioactive elements [region (3) in Figure 1B], I calculate the power-law formula between *N* and *Z* using all data [both regions (1) and (2) in Figure 1B] at the double-logarithmic scale; the relation of *N* versus *Z* for all data at the double-linear scale [both regions (1) and (2) in Figure 1A] is also computed for comparison. The AREs of the predictions for $\bar{N}$ , *N*_rad_, and both $\bar{N}$ and *N*_rad_ obtained using the power law (*N* = 0.73 × *Z* ^1.16^) are 90%, 11%, and 88% lower, respectively, than the linear relation at the double-linear scale (*N* = 1.61 × *Z* − 9.01), and the RMSEs of the predictions for $\bar{N}$ , *N*_rad_, and both $\bar{N}$ and *N*_rad_ obtained using the power law are 59%, 17%, and 23% lower, respectively, than the linear relation (Table 2). These results again support the above conclusion that the predictions for *N* by power laws are more accurate than those by linear relations obtained on the Segrè chart.

## 4 Discussion

As quantum systems, nuclei’s behaviors can be depicted with the nonrelativistic Schrödinger equation (Olavo, 1999; Schleich et al., 2013); within nuclei, how triple quarks gather to form protons and neutrons can be interpreted by quantum chromodynamics (QCD) (Marciano and Pagels, 1978; Greiner et al., 2007; Group et al., 2022). However, it is well known that describing the features of isotopes does not need to explicitly include quarks in theoretical models; instead, having protons and neutrons in these models is sufficient to predict a variety of properties of isotopes (Blatt and Weisskopf, 1991; Thomson, 2013). For example, the mass of a nucleus can be estimated from its number of protons and neutrons using the Weizsäcker mass formula (Weizsäcker, 1935), which is a refined form of the liquid drop model for the binding energy of nuclei (Gamow, 1930). Moreover, neutrons also affect the stability of nuclei and isotopes’ decay (Mueller and Sherrill, 1993; Pfützner et al., 2012). In a stable nucleus, valence neutrons are well bound; the corresponding wavefunction decays rapidly when the valence neutron is outside the limit of stability (Blatt and Weisskopf, 1991; Mueller and Sherrill, 1993). In contrast, within an unstable nucleus, valence neutrons are loosely bound and live in the classically forbidden region; the corresponding wavefunction possesses a long tail (Blatt and Weisskopf, 1991; Mueller and Sherrill, 1993). For a specific element (with a fixed number of protons), the lifespans of its isotopes are influenced by the number of neutrons (Thomson, 2013; Martin and Shaw, 2017). Therefore, the number of neutrons offers a window through which to predict the long-lived isotopes of unknown radioactive elements.

Statistical analyses in this study show that power laws exist between the number of neutrons and the number of protons in both stable isotopes of non-radioactive elements and long-lived isotopes of radioactive elements. Power laws have been identified in a variety of natural systems, such as the evolutionary processes of life over geological time scales (Raup, 1986; Shang, 2024), the distributions of amino acids and expressed genes in various organisms and tissues (Furusawa and Kaneko, 2003; Mora and Bialek, 2011), and the degradation rate versus age of organic matter in ecosystems (Middelburg, 1989; Shang, 2023). However, the specific reasons for many observed power-law patterns are not well understood (Clauset et al., 2009; Alstott et al., 2014). Similarly, why the coefficient and exponent in the power-law relation between *N* and *Z* (Figure 1B, Figure 3; Table 1) take those specific values remain unknown. Moreover, the underlying physical mechanisms responsible for the emergence of these power laws may not be readily interpreted by our current knowledge of nucleons. It is well known that the binding energy of a nucleus derives from the strong interaction, which is described by QCD (Marciano and Pagels, 1978; Greiner et al., 2007; Group et al., 2022). Nevertheless, the (low-energy) QCD is not at the stage where we can use it to obtain comprehensive understating of nucleons (Marciano and Pagels, 1978; Greiner et al., 2007; Group et al., 2022). From the perspective of statistical mechanics, power laws are usually attributed to self-organized criticality, a concept that was originally suggested by Bak, Tang, and Wiesenfeld (Bak et al., 1987). Self-organized criticality refers to the phenomenon that the internal interactions of a system organize itself into states where power laws appear (Bak et al., 1987, 1988). However, studies have shown that power-law patterns are an emergent property of self-organized criticality and do not necessarily originate from the latter (Solow, 2005; Clauset et al., 2009; Marković and Gros, 2014). Whether the power-law pattern observed in this study (Figure 1B, Figure 3; Table 1) originates from certain mechanisms related to self-organized criticality in nuclei requires further investigation.

The power laws shown in this work provide better prediction for the number of neutrons in stable isotopes of non-radioactive elements or long-lived isotopes of radioactive elements than the linear relation on the Segrè chart (Tables 1, 2). The power law, *N* = 0.73 × *Z* ^1.16^, which is obtained using all data (i.e., both $\bar{N}$ and *N*_rad_ versus *Z*) at the double-logarithmic scale (Table 2), therefore, may be applied to predict *N* values of long-lived isotopes of unknown radioactive elements with *Z* ≥ 119 [region (3) in Figure 1B]. For example, with this mathematical formula, one may expect that the mean number of neutrons in long-lived isotopes of the unknown radioactive element with *Z* = 120 would be about 188; the region surrounding this point, (120, 188), on the plot of *N* versus *Z* at the double-logarithmic scale would be the island of stability for the isotopes of this unknown radioactive element. However, one should note that predicting the maximum number of neutrons that can exist in a stable/long-lived nucleus, which has been recognized as a difficult task for *ab initio* many-body theories (Ring and Schuck, 2004; Hergert, 2020), is beyond the ability of the power-law relation presented in this work.

Studies have suggested that the neutron-proton asymmetry significantly influences the stability of a nucleus; as the value of *Z* increases, the stability of a nucleus decreases due to the growth of Coulomb repulsion (Blatt and Weisskopf, 1991; Martin and Shaw, 2017). This implies that the power-law pattern presented here probably will disappear when *Z* exceeds a certain large, critical value. Actually, power laws observed in natural systems often vanish at some critical points (Schroeder, 2009; Bak, 2013). Although this appears daunting, no available clue shows that the disappearance of the power-law pattern occurs immediately when *Z* ≥ 119. Therefore, the mathematical formula, *N* = 0.73 × *Z* ^1.16^, may still provide empirical guidance for probing the long-lived isotopes of unknown radioactive elements. Future discovery of new radioactive elements will offer further validation for the predictive ability of the power-law relation revealed in this study.

## Data Availability

The original contributions presented in the study are included in the article/Supplementary Material, further inquiries can be directed to the corresponding author.
